# Meta-analysis of differences in neutrophil to lymphocyte ratio between hypertensive and non-hypertensive individuals

**DOI:** 10.1186/s12872-023-03304-w

**Published:** 2023-06-03

**Authors:** Shirin Sarejloo, Moein Dehesh, Mobina Fathi, Monireh Khanzadeh, Brandon Lucke-Wold, Arshin Ghaedi, Shokoufeh Khanzadeh

**Affiliations:** 1 Cardiovascular Research Center, Shiraz University of Medical Sciences, Shiraz, Iran; 2 Johns Hopkins University, Baltimore, USA; 3 Shahid Beheshti University of Medical Sciences, Tehran, Islamic Republic of Iran; 4 Geriatric & Gerontology Department, Medical School, Tehran University of Medical and Health Sciences, Tehran, Iran; 5 Department of Neurosurgery, University of Florida, Gainesville, USA; 6 Student Research Committee, School of Medicine, Shiraz University of Medical Sciences, Shiraz, Iran; 7 Tabriz University of Medical Sciences, Tabriz, Islamic Republic of Iran

**Keywords:** Hypertension, Inflammation, Neutrophil to lymphocyte ratio, Dipper, Meta-analysis

## Abstract

This study systematically reviewed the evidence regarding differences in the neutrophil to lymphocyte ratio (NLR) level between hypertensive and normotensive individuals as well as between patients with dipper and non-dipper hypertension (HTN). PubMed, Scopus, and Web of Science databases were systematically searched up to 20 December 2021. This was done without any limitation with regard to date, publication, or language. Pooled weighted mean differences (WMD) with 95% confidence intervals (95% CI) were reported. We assessed the quality of studies based on the Newcastle–Ottawa Scale (NOS). In total, 21 studies were included in our study. There was a significant increase in NLR levels for the hypertensive group in comparison to the control group (WMD = 0.40, 95%CI = 0.22–0.57, *P* < 0.0001). In addition, the NLR levels were higher in the non-dipper than in the dipper group (WMD = 0.58, 95%CI = 0.19–0.97, *P* = 0.003). Our findings showed that hypertensive patients had higher level of NLR than normotensive individuals.

## Introduction

Hypertension, a globally prevalent noncommunicable disease, has gained prominence in recent years as medical researchers have discovered some of the inflammatory components that underpins its etiology. In addition, several complications of hypertension, such as retinopathy, neuropathy, and cardiomyopathy, have been linked to the inflammatory response that develops in the arterial walls over time due to consistently elevated pressures. In addition, there is a large volume of published studies reporting the elevated level of inflammatory biomarkers in HTN patients. These studies have shown that inflammation in HTN occurs not only in the arterial walls, but also throughout the whole body. HTN can be divided into two groups, including the dipper and non-dipper groups. In patients with dipper HTN, systolic and diastolic blood pressure dropped by more than 10% during sleep. This diurnal pattern is thought to be a normal variant. Patients whose blood pressure does not show this diurnal pattern are referred to as "non-dippers." Non-dippers have a higher risk of cardiovascular disease and target organ damage than dippers [1, 2]. It is very important to find responsible pathophysiological conditions which may be the cause of this risk rise. Some research teams speculated that the inflammatory process plays a role in this phenomenon; so they compared the inflammatory biomarkers between these two groups.

A large and growing body of literature has investigated the role of inflammatory biomarkers and cytokines in HTN. However, in recent years, there has been increase interest in simple hematologic biomarkers such as the neutrophil to lymphocyte ratio (NLR) and platelet to lymphocyte ratio (PLR). Blood NLR is a simple marker for chronic low-grade inflammation that can be obtained easily from a differential blood count [3]. Neutrophils and lymphocytes are key immune system cellular components. Neutrophils are a type of innate immunity cell that can produce chemokines, cytokines, vascular endothelial growth factor (VEGF), and matrix metalloproteinase to reinforce the initial line of the immune system response. Lymphocytes, which are adaptive immunity cells, are also fine tuned controllers of this particular immune response [4]. As neutrophils and lymphocytes interact with each other, their ratio and sheer numbers have an impact on the immune response amplitude [5]. Increased neutrophil numbers, in particular, decrease lymphocyte activity [6, 7]. Recently, the NLR has emerged as an indicator of systemic inflammation in a variety of disorders including cancer [8], neurologic disorders [9], and infectious diseases [10]. It has been used as an independent prognostic biomarker in various clinical settings, predicting major mortality, morbidity, and long-term survival [11–14]. In the context of cardiovascular diseases, NLR is an emerging marker in patients with heart failure [15], acute coronary syndrome [16], stable coronary artery disease [17–20], and for patients undergoing percutaneous coronary interventions [21] or coronary artery bypass grafting [22]. In addition, there is a large volume of published studies describing the role of NLR in HTN. The majority report that hypertensive patients had elevated levels of the NLR compared to normotensive individuals and more specifically that non-dippers had an elevated level of NLR compared to dippers [23–34]. However, some studies showed no differences [35–43]. Although extensive research has been carried out on the role of NLR in HTN, no single study exists which reviews the available evidence in order to draw a single result from contradictory findings.

This study systematically reviewed the evidence regarding the differences in the NLR level between hypertensive and normotensive individuals as well as between patients with dipper and non-dipper HTN. The goal was to develop an understanding of the pathophysiology of HTN and explain the risk rise of cardiovascular events in dippers compared to non-dippers using the NLR.

## Material and method

### Search strategy and study selection

We conducted this meta-analysis in accordance with the Preferred Reporting Items for Systematic Reviews and Meta-Analysis (PRISMA) guidelines [44]. PubMed, Scopus, and Web of Science databases were systematically searched up to 20 December 2021 using the following keywords: ((neutrophil AND lymphocyte AND ratio) OR neutrophil-to-lymphocyte OR NLR) AND Hypertension. No date or language restrictions were considered. In addition, we scanned the reference lists of related articles manually to find potentially missing or additional eligible studies.

The inclusion criteria based on the PICOS principle were as follows.

- Population: Patients with HTN (either primary or secondary HTN) in first analysis AND patients with non-dipper HTN in the second analysis
- Intervention (Exposure): High NLR
- Control: Healthy control in first analysis AND patients with dipper HTN in the second analysis
- Outcomes: Diagnostic role of NLR
- Studies: case–control, cross-sectional, and cohort studies

If the study did not report the level of NLR as a mean or standard deviation (SD), Wan et al.’s method was used to calculating the estimated values [45]. In this study, they discuss different approximation methods for the estimation of the sample mean and SD and proposed some new estimation methods to improve the existing literature. They conclude their work with a summary table (an Excel spread sheet including all formulas) that serves as a comprehensive guidance for performing meta-analysis for different situations. We used this same Excel sheet in our study.

We excluded the incomplete studies and abstracts, reviews, case reports, and animal studies. Two authors independently selected the articles for final inclusion according to these criteria, and if discrepancies existed, a third author resolved any disagreements.

### Data extraction

The extracted data were as follows: (1) first author; (2) country of origin; (3) year of publication; (4) study design; (5) number of cases and controls; (6) NLR level from cases and controls; (7) drug history; (8) mean age; (9) gender.

### Data synthesis and analysis

Pooled weighted mean difference (WMD) with 95% confidence interval (95% CI) was used to assess the differences in NLR levels between the patients with HTN and the controls or between dipper and non-dipper HTN patients. Because different studies used similar methods to measure the NLR, the unit of NLR among included studies was recorded the same. We assessed the quality of studies based on the Newcastle–Ottawa Scale (NOS) [46], with a maximum grade of nine for each study. Heterogeneity across included studies was calculated using I ^2^ statistics and Q test. The I ^2^ values showed serious (I ^2^ = 75–100%), high (I ^2^ = 50–74.9%), moderate (I ^2^ = 25–49.9%), low (I ^2^ = 0.1–24.9%), and no (I ^2^ = 0) heterogeneity. Furthermore, a significant Q-statistic showed heterogeneity among studies. If heterogeneity was high or serious (I2 ≥ 50%), we used the random-effect model; otherwise, we used the fixed-effect model. In addition, we used meta-regression and subgroup analysis to explore source of heterogeneity. Subgroup analysis was stratified by sample size. The small study was defined as studies with sample size ˂ 150, and studies with ≥ 150 patients were considered large studies. Egger’s and Begg’s tests and funnel plots were used to determine the publication bias. STATA 12.0 software (Stata Corporation, College Station, TX, USA) was used in data analyses. A 2-sided *P* < 0.05 was considered statistically significant.

### Certainty of evidence

Two authors determined the certainty of evidence using the GRADE (Grading of Recommendations Assessment, Development and Evaluation) approach for two outcomes (HTN and non-dipper HTN).

## Results

**Fig. 1:**
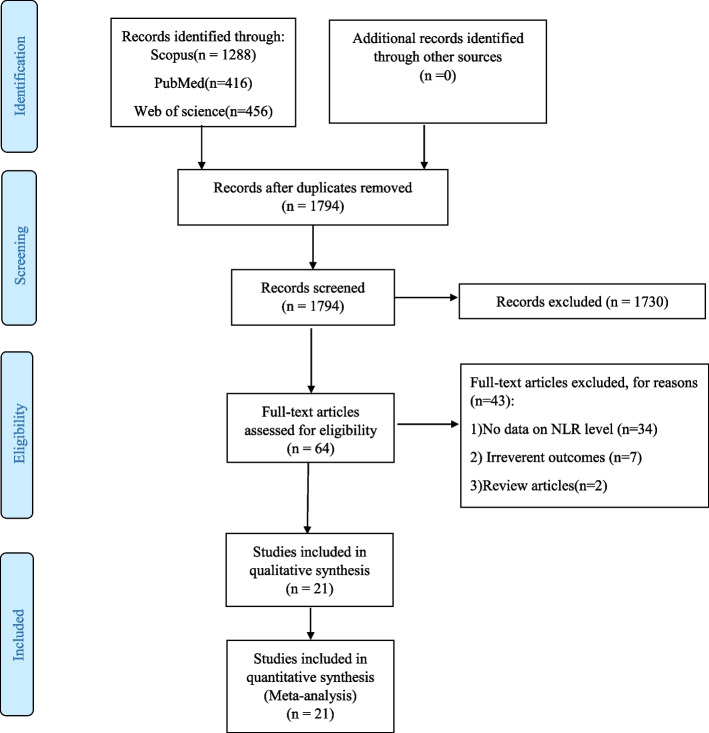
Flow chart of search and study selection

The literature search gave a total of 2165 articles. After emitting the duplicates, 1794 remained. Among them, 64 were found to be relevant in initial evaluation based on title and abstract. An additional 34 studies were excluded due to lack of data on NLR level, seven due to irrelevant outcomes, and two because they were review articles. Finally, 21 studies [23–43] investigating the association between NLR and HTN were included in this meta-analysis **(**Fig. 1**)**.

### Characteristics of the included studies

Of the 21 studies included in this meta-analysis [23–43], six were case–control studies [25, 33, 35, 41–43], and 15 were cross-sectional studies [23, 24, 26–32, 34, 36–40]. Concerning document language, all of the documents were in English. Overall 2396 patients with HTN and 1016 normotensive controls were enrolled in the selected studies. The general characteristics of the included studies is shown in Tables 1 and 2. NLR levels in hypertensive patients were compared with those of normotensive controls in 17 studies. In terms of sample size, there were nine large studies [25–32, 36] and eight small studies [33, 34, 38–42]. In addition, eight studies compared patients with dipper and non-dipper HTN utilizing the NLR [23, 24, 26, 28, 31, 32, 37, 43].

**Table 1:** General characteristics of included studies

Author name	Year	Design	Hypertensive patients						Normotensive controls		NOS score
			Total		Dipper		Non-dipper				
			N	NLR	N	NLR	N	NLR	N	NLR	
Demir	2013	Cross-sectional	80	-	50	1.80 ± 0.52	30	3.10 ± 0.95	-	-	7
Sunbul	2013	Cross-sectional	166	-	83	1.80 ± 0.50	83	2.30 ± 0.90	-	-	8
Kilicaslan	2014	Cross-sectional	81	2.31 ± 0.90	39	1.88 ± 0.60	42	2.71 ± 1.18	69	2.13 ± 0.87	6
Mehmood	2014	Case–control	30	1.94 ± 0.63	-	-	-	-	30	1.72 ± 0.52	6
Yayla	2014	Cross-sectional	101	2.49 ± 0.77	-	-	-	-	54	1.80 ± 0.65	8
Belen	2015	Case–control	100	2.63 ± 0.51	-	-	-	-	50	1.87 ± 0.35	6
Kim	2016	Cross-sectional	535	2.46 ± 2.17	269	2.02 ± 1.32	266	2.91 ± 3.04	112	1.75 ± 1.77	7
Unamba	2017	Cross-sectional	144	1.35 ± 0.80	-	-	-	-	72	1.23 ± 0.60	7
Wang	2017	Cross-sectional	217	1.91 ± 0.68	-	-	-	-	132	1.65 ± 0.53	8
Bozduman	2018	Cross-sectional	91	2.80 ± 0.78	35	2.00 ± 0.60	56	3.30 ± 0.90	108	1.85 ± 0.55	8
Derya	2018	Cross-sectional	80	3.14 ± 2.16	-	-	-	-	80	1.89 ± 0.55	7
Skrzypczyk	2018	Cross-sectional	54	2.06 ± 1.30	-	-	-	-	20	1.91 ± 0.80	9
Srinivasagopalan	2018	Case–control	80	2.11 ± 0.74	-	-	-	-	40	1.64 ± 0.71	8
Tek	2018	Cross-sectional	95	-	47	1.81 ± 0.69	48	1.91 ± 0.69	-	-	7
Yousif	2018	Cross-sectional	91	2.37 ± 1.37	-	-	-	-	31	2.04 ± 0.82	7
Atmaca	2019	Cross-sectional	47	2.21 ± 1.28	-	-	-	-	47	2.07 ± 1.12	9
Cetin	2019	Cross-sectional	89	1.66 ± 0.83	28	1.86 ± 1.09	61	1.58 ± 0.72	64	1.38 ± 0.54	7
Balan	2020	Case–control	26	1.75 ± 0.68	-	-	-	-	38	1.76 ± 0.95	8
Berillo	2020	Case–control	16	1.60 ± 0.20	-	-	-	-	15	1.80 ± 0.40	8
Chotruangnapa	2021	Case–control	208	-	104	1.86 ± 0.90	104	1.87 ± 0.70	-	-	8
Hou	2021	Cross-sectional	65	2.18 ± 1.12	-	-	-	-	54	1.68 ± 0.75	8

*N* Number, *NLR* Neutrophil to lymphocyte ratio, *NOS* Newcastle–Ottawa Scale

**Table 2:** Demographic characteristics of included studies

Author name	Year	Country	Mean age	Male (%)	Mean BMI	Smoking(%)	Diabetes	Method used for NLR measurement	Definition of HTN group	Drug history in study group
Demir	2013	Turkey	50.6	57%	28.4	20%	0%	Blood samples were drawn by venipuncture to perform routine blood chemistry	SBP ≥ 140 or DBP ≥ 90 or taking anti-hypertensive drugs	Betablocker:15.9%; CCB:25.6%; ARB:18.6%; ACE inhibitor:35.5%;Diuretic:70.2%
Sunbul	2013	Turkey	52.3	49%	_	19%	14%	Complete blood counts were obtained at the time of admission	SBP %140 mmHg and/or DBP %90 mmHg, previously diagnosed hypertension, or use of any antihypertensive medications	ND
Kilicaslan	2014	Turkey	57.3	49%	_	_	0%	Blood samples were drawn in the morning after a 20-min rest following a fasting period of 12 h	Three clinic BP measurements (> 140/90 mmHg) taken at 1-week intervals in the absence of any previous antihypertensive treatment	ND
Mehmood	2014	Pakistan	47.90	100%	26.76	0%	0%	Complete blood counts were obtained at the time of admission	According to JNC-VII report	ND
Yayla	2014	Turkey	52.4	46%	25.1	28.7%	0%	Blood samples were taken from the antecubital vein after 12 h of fasting during the initial admission day	SBP ≥ 140 or DBP ≥ 90	ND
Belen	2015	Turkey	62.06	40%	27.4	0%	0%	Blood samples were withdrawn from an antecubital vein, with atraumatic venipuncture, in the morning after a 12-h fasting period	A mean ambulatory daytime BP of 135/85 mm Hg	Beta-blocker:36%; CCB:40%; ARB: 32%; Diuretic:34%
Kim	2016	Korea	51.6	50%	25.4	7%	7%	Complete blood cell counts were obtained at the first visit	Average daytime BP higher than 135/85 mm Hg and the average nighttime BP above 120/70 mm Hg	Beta-blocker:15.14%; CCB:20%; ARB:20.93%; ACE inhibitor:20.93; Diuretic:7%
Unamba	2017	Nigeria	51.4	45%	29.5	0%	0%	Fasting blood samples were collected for full blood count	SBP ≥ 140 and/or DBP ≥ 90	ND
Wang	2017	China	47.54	61%	27.74	_	14.13%	Venous blood samples were drawn from the antecubital vein following a 12-h fasting period	SBP > 140 mm Hg and DBP > 90 mm Hg	Beta-blocker:36.96%; CCB:44.52%; ACE inhibitor:56.52%
Bozduman	2018	Turkey	54.4	57%	28.3	23.2%	25%	The hematologic and biochemical samples after 10–12 h fasting were collected	SBP ≥ 140 or DBP ≥ 90	ND
Derya	2018	Turkey	43	54%	29.0	31%	0%	After the first admission and following a 12-h fast, blood samples were obtained from the antecubital vein	24-h mean SBP of ≥ 130 mmHg and/or 24-h mean DBP ≥ 80 mmHg	ND
Skrzypczyk	2018	Poland	15.12	69%	25.55	_	_	_	Systolic and/or diastolic pressure ≥ 95th percentile for sex, age, and height during 24 h according to AHA guidelines	ND
Srinivasagopalan	2018	India	44.08	_	_	0%	_	The blood sample was drawn under aseptic precautions in an anticoagulant containing vial	SBP ≥ 140 mmHg and DBP ≥ 90 mmHg	ND
Tek	2018	Turkey	46.64	51%	_	_	0%	Patient’s blood samples were collected at the same day of ABPM records	Daytime SBP > 135 mmHg and DBP > 85 mmHg in ambulatory blood pressure monitoring	ND
Yousif	2018	Turkey	54.8	25%	33.4	3.3%	0%	Peripheral venous blood samples have been collected after a 12 h fasting	Mean arterial pressure140/90 mmHg on at least 3 separate occasions, or receiving antihypertensive treatment	ND
Atmaca	2019	Turkey	72.7	40%	_	0%	_	Peripheral venous blood samples were drawn from all subjects after 12 h of hunger at sitting position from antecubital vein	At least 15-years of hypertension history. The BP ranges were not declared	ND
Cetin	2019	Turkey	11.3	49%	23	_	_	The clinical and laboratory information were obtained by electronic medical records	The mean systolic or diastolic ambulatory BP were ≥ the 95th percentiles for age, gender, and height during either the sleep or awake period	ND
Balan	2020	Romania	59.04	47%	_	_	_	_	SBP ≥ 135 mmHg and/or DBP ≥ 85 mmHg	ND
Berillo	2020	France	59	56%	26	_	0%	Blood samples were collected in the morning under fasting conditions	BP ≥ 135/85 mm Hg, or treatment with antihypertensive medications for at least 6 months	Beta-blocker:25%;CCB: 38%;ARB: 81%; ACE inhibitor: 81%;Diuretic:44%
Chotruangnapa	2021	Thailand	63	32%	24.2	0%	32.7%	Complete blood counts were obtained at the nearest time of performing 24 h ABMP	1) office systolic blood pressure (SBP) > 140 mmHg and/or diastolic blood pressure (DBP) > 90 mmHg or 2) home SBP > 135 mmHg and/or DBP > 85 mmHg or 3) ABPM: daytime mean SBP > 135 mmHg and/or DBP > 85 mmHg or night-time mean SBP > 120 mmHg and/or DBP > 70 mmHg or 24 h mean SBP > 130 mmHg and/or DBP > 80 mmHg or received anti-hypertensive medications	Beta-blocker:24.7%; CCB: 45.7; ARB: 31.7%; ACE inhibitor:16.3%;Diuretic:13.5%
Hou	2021	China	12.37	74%	_	_	_	Blood was obtained from an antecubital venous catheter after 10–12 h of night fasting	Systolic and/or diastolic pressure ≥ 95th percentile for sex, age, and height according to the reference value of the Chinese Child Blood Pressure References Collaborative Group	ND

*ND* Not Declared, *CCB* Calcium channel blocker, *ACE* Angiotensin-converting enzyme, *ARB* Angiotensin receptor blockers

### Differences between hypertensive and normotensive individuals in NLR level

**Fig. 2:**
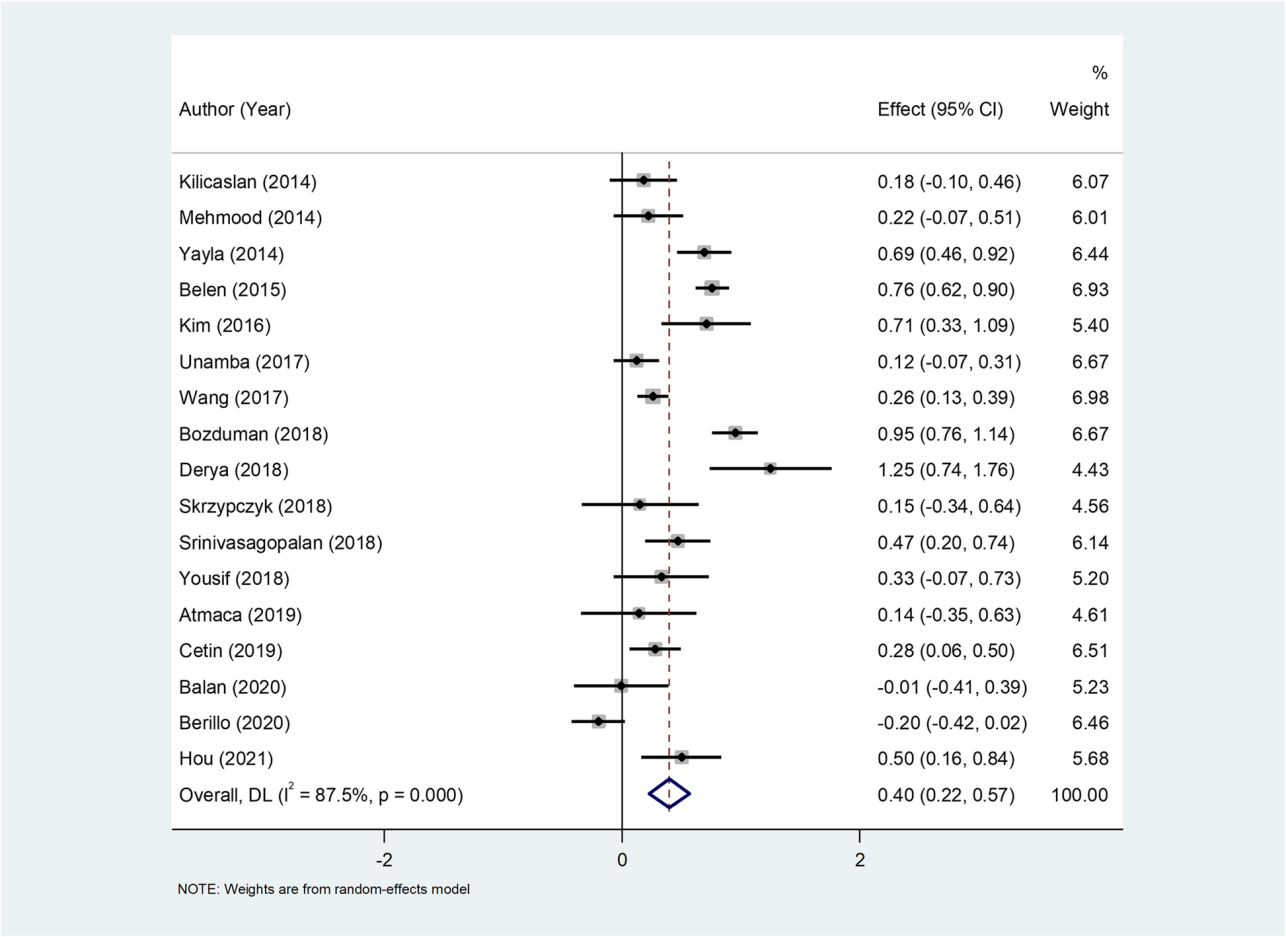
Metaanalysis of differences in NLR levels between patients with HTN and normotensive controls

NLR level differences between HTN patients and normotensive controls were investigated in 17 studies, including 1847 patients and 1016 controls. The pooled results showed that there was a significant increase of NLR levels in the hypertensive group in comparison to the control group (WMD = 0.40, 95%CI = 0.22–0.57, *P* < 0.0001, Fig. 2). There was a significant heterogeneity (I ^2^ = 87.5%, *p* < 0.001); so we used random- effect model. However, the certainty of the evidence was low (Table 3).

**Table 3:** GRADE^a^ Evidence Profile for cohort studies of the neutrophil to lymphocyte ratio in post-stroke infection

Certainty assessment							№ of patients		Certainty	Importance
**№ of studies**	**Study design**	**Risk of bias**^**b**^	**Inconsistency**^**c**^	**Indirectness**	**Imprecision**^**d**^	**Publication bias**^**e**^	**Participants, n**	**Cases, n**		
**HTN**										
17	observational studies	not serious	very serious	not serious	not serious	none	2863	1847	⨁◯◯◯ Very low	CRITICAL
**Non-dipper HTN**										
8	observational studies	not serious	Very serious	not serious	not serious	none	1343	690	⨁◯◯◯ Very low	CRITICAL

^a^Grading of Recommendations Assessment, Development and Evaluation

^b^Risk of bias based on Newcastle–Ottawa Scale

^c^When I^2^ was < 30% inconsistency considered as Not serious limitation, > 50 considered as serious and more than 75% considered as very serious limitation

^d^Serious limitations when there was fewer than 400 participants for each outcome and very serious limitations when there was fewer than 300 participants for each outcome

^e^Funnel plot revealed no asymmetry; neither test of publication bias approached *sssss* < 0.10

**Fig. 3:**
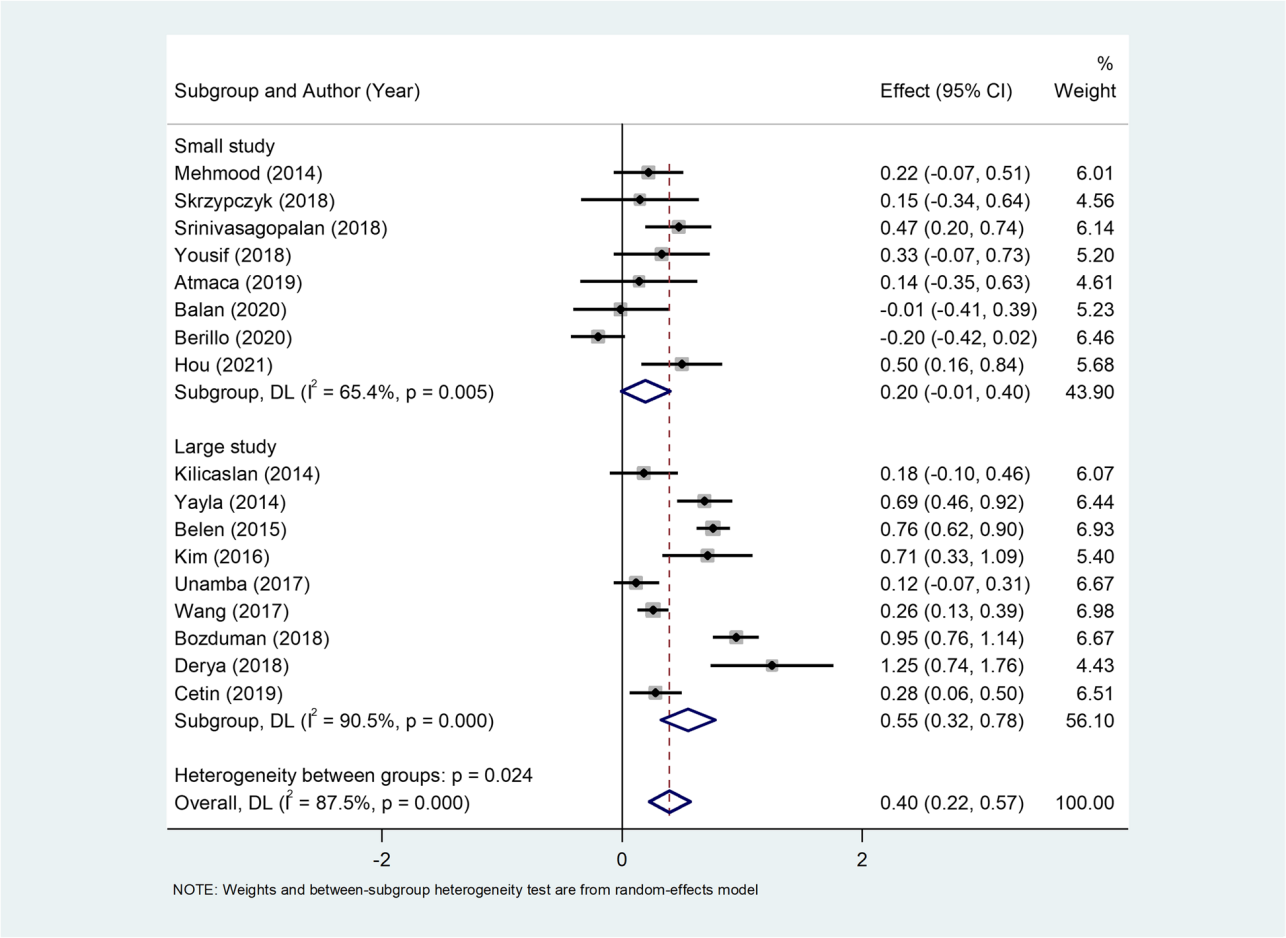
Subgroup analysis of differences in NLR levels between patients with HTN and normotensive controls according to sample size

In the subgroup analysis according to sample size, there were nine large studies [25–32, 36], including 1438 hypertensive and 741 normotensive individuals. There were eight small studies [33, 34, 38–42] with 409 hypertensive and 275 normotensive individuals. Patients with HTN had higher levels of NLR in either small (WMD = 0.20, 95%CI = -0.01–0.40, *P* = 0.06) or large studies (WMD = 0.55, 95%CI = 0.32–0.78, *P* < 0.001) in comparison to normotensive individuals (Fig. 3).

In the metaregression analysis, there was no significant effect of total sample size (B < -0.001, adjusted R^2^ = -8.89, *p* = 0.68), publication year (B = -0.08, adjusted R^2^ = 4.06, *p* = 0.18), NOS score (B = 0.16, adjusted R^2^ = 0.0%, *p* = 0.95), gender (B = -8.21, adjusted R^2^ = 87.12, *p* = 0.83), mean age of cases (B = 0.001, adjusted R^2^ = 86.17, *p* = 0.86), smoking (B = 0.01, adjusted R2 = 7.11, p = 0.24), diabetes (B = 0.02, adjusted R^2^ = 3.62, *p* = 0.27), NOS score (B = -0.10, adjusted R^2^ = -4.54, *p* = 0.45), or BMI (B = 0.01, adjusted R^2^ = -12.40, *p* = 0.86). In addition, use of beta-blocker (B = 0.04, adjusted R^2^ = -20.85, *p* = 0.52), calcium channel blockers (CCB) (B = 0.01, adjusted R^2^ = 95.35, *p* = 0.85), Angiotensin receptor blockers (ARB) (B = -0.02, adjusted R^2^ = 97.68, *p* = 0.50) and diuretics (B = -0.009, adjusted R^2^ = 97.23, *p* = 0.89) had no effect on the NLR; so they could not be the source of heterogeneity. However, use of angiotensin-converting enzyme (ACE) inhibitors (B = -0.01, adjusted R^2^ = 84.87, *p* = 0.44) had significant effect on NLR; so it could be the source of heterogeneity.

### Differences between patients with dipper and non-dipper HTN in NLR level

**Fig. 4:**
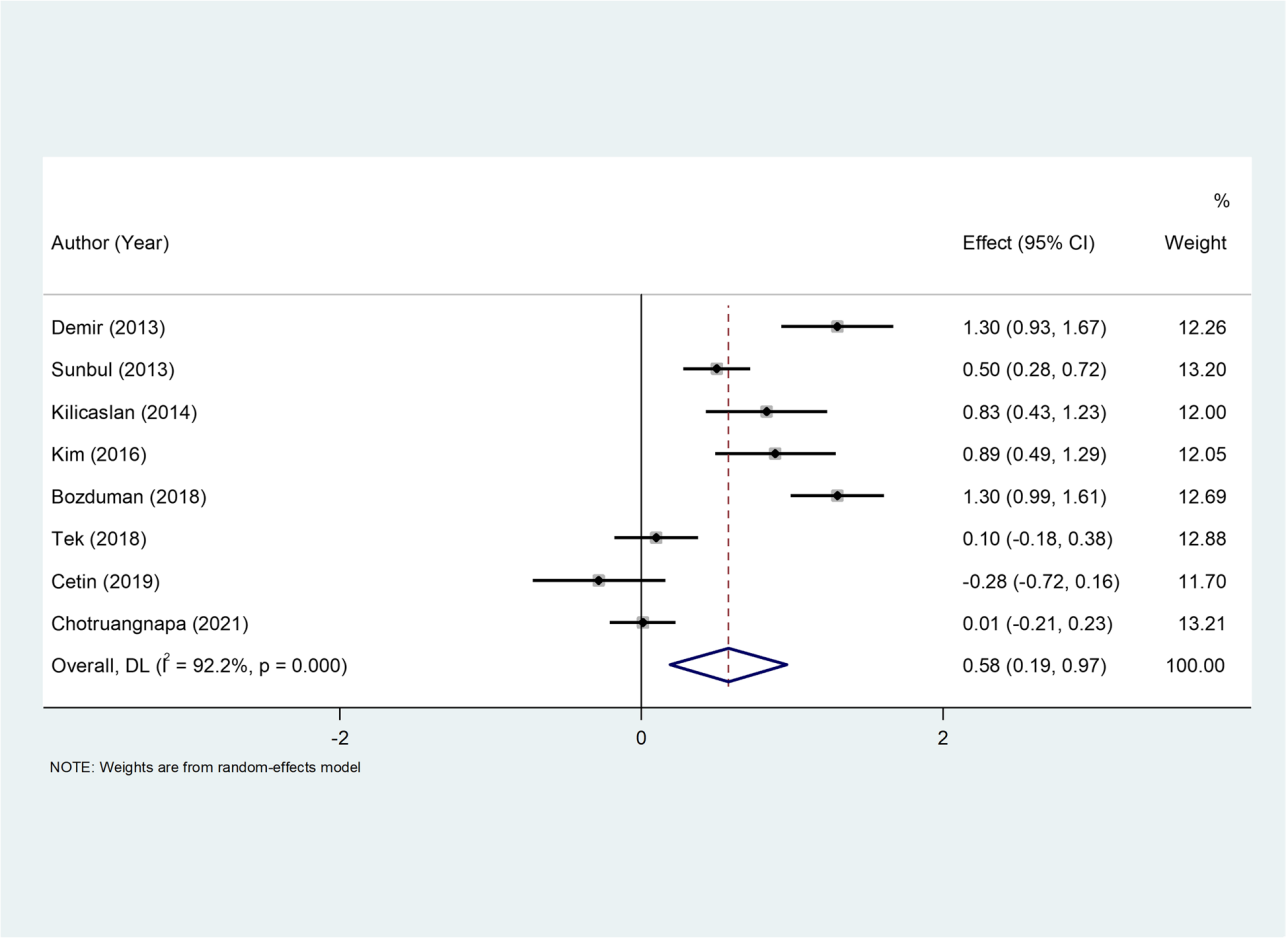
Metaanalysis of differences in NLR levels between patients with dipper and non-dipper HTN

The pooled result of eight studies [23, 24, 26, 28, 31, 32, 37, 43] including 655 patients with dipper HTN and 690 patients with non-dipper HTN in NLR levels showed that the NLR levels were higher in non-dipper than in the dipper group (WMD = 0.58, 95%CI = 0.19–0.97, *P* = 0.003, Fig. 4). However, there was a significant heterogeneity (I ^2^ = 92.2%, *p* < 0.001); so we used a random-effect model. According to GRADE method, the certainty of the evidence was low (Table 3).

**Fig. 5:**
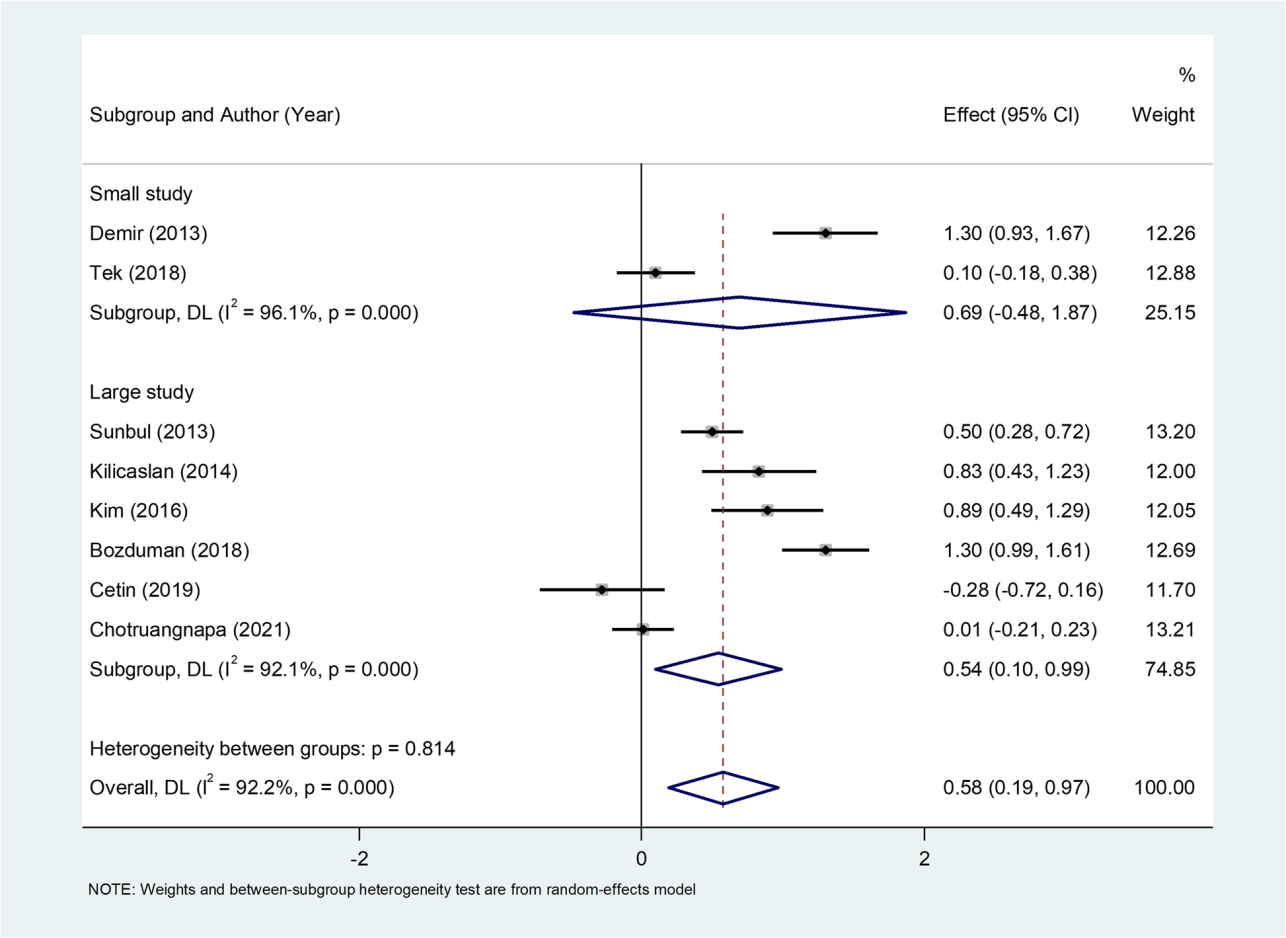
Subgroup analysis of differences in NLR levels between patients with dipper and non-dipper HTN, according to sample size

In the subgroup analysis according to sample size, there were six large studies, including 655 patients with dipper HTN and 690 patients with non-dipper HTN. There were two small studies with 655 patients with dipper HTN and 690 patients with non-dipper HTN. Patients with non-dipper HTN had higher levels of NLR in large studies (WMD = 0.54, 95%CI = 0.10–0.99, *P* = 0.01), but not in small studies (WMD = 0.69, 95%CI = -0.48–1.87, *P* = 0.24) when comparing to patients with dipper HTN (Fig. 5).

In the metaregression analysis, we found that smoking (B = 0.06, adjusted R^2^ = 74.68, *p* = 0.05) and BMI (B = 0.39, adjusted R^2^ = 100, *p* = 0.004) could be a source of heterogeneity. However, there was no significant effect of total sample size (B = -0.0001, adjusted R^2^ = -15.53, *p* = 0.66), publication year (B = -0.14, adjusted R^2^ = 25.86, *p* = 0.13), NOS score (B = 0.03, adjusted R^2^ = -18.87, *p* = 0.93), gender (B = 0.06, adjusted R^2^ = 33.87, *p* = 0.08), mean age of cases (B = 0.02, adjusted R^2^ = 5.28, *p* = 0.28), smoking (B = 0.06, adjusted R^2^ = 74.68, *p* = 0.05), diabetes (B = -0.008, adjusted R^2^ = -18.68, *p* = 0.71), and BMI (B = 0.39, adjusted R^2^ = 100, *p* = 0.004) on the association between NLR and HTN; so they could not be the source of heterogeneity.

### Publication bias

**Fig. 6:**
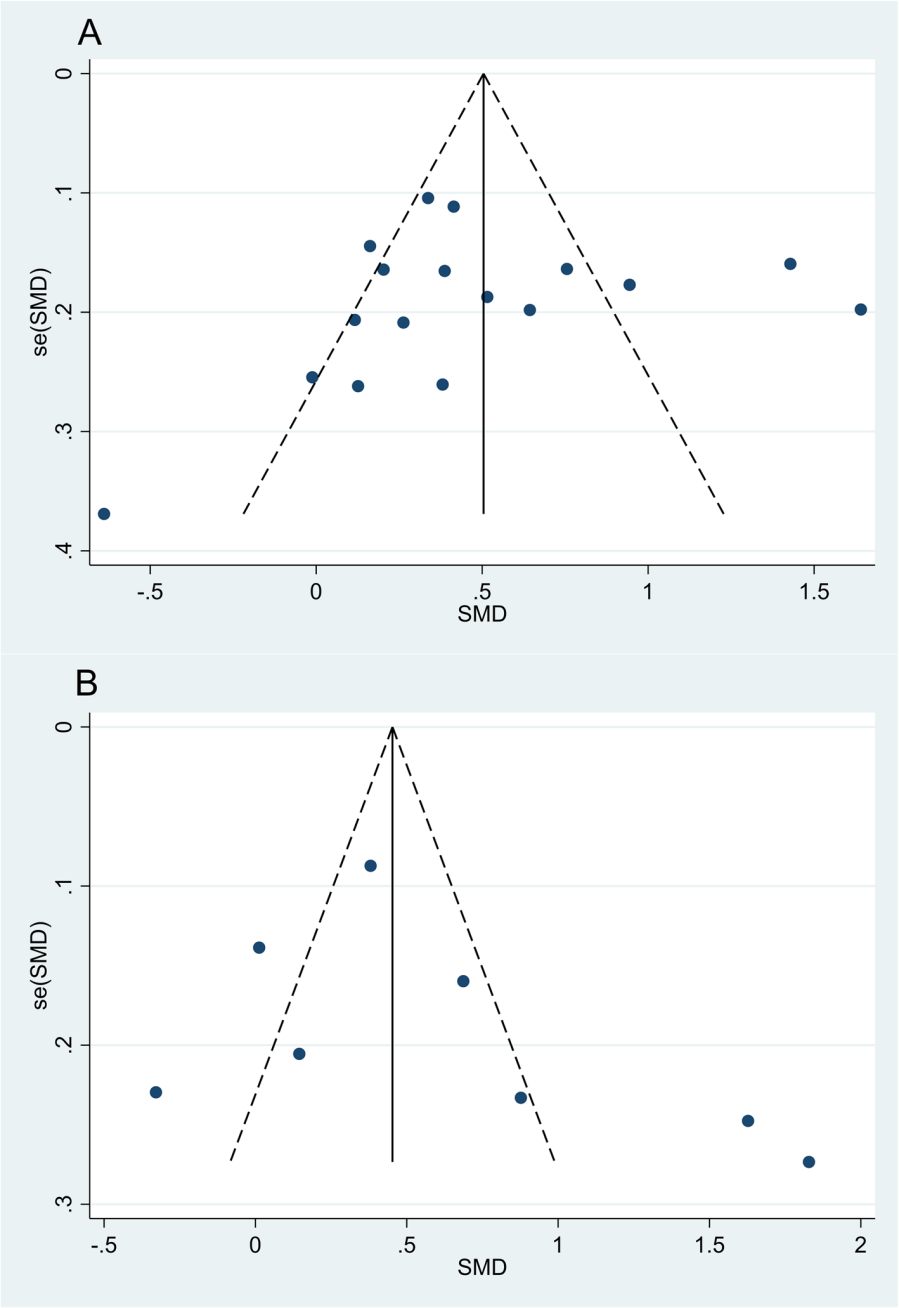
Funnel plot assessing the potential publication bias; **A **Studies on the differences in NLR levels between patients with HTN and normotensive controls; **B **studies on the differences in NLR levels between patients with dipper and non-dipper HTN

As seen in Fig. 6, the funnel plots are asymmetrical and suggest that publication bias may exist. However, none of the statistical methods for subgroup analysis found such differences in NLR levels between patients with HTN and normotensive controls (Egger’s test *P* = 0.09, Begg’s test *P* = 0.08), and between patients with dipper and non-dipper HTN (Egger’s test 0.13, Begg’s test *P* = 0.10).

## Discussion

The exact etiology which underlies HTN, a known risk factor for cardiovascular disease, is still unclear [31, 47]. In this meta-analysis, we systematically reviewed papers working on the NLR level in normotensive individuals and dipper and non-dipper hypertensive patients. Our results indicate that NLR level was significantly higher in hypertensive individuals compared with normotensive individuals. Also, it has been demonstrated that non-dipper hypertensive patients have increased NLR levels in comparison to dipper hypertensive patients. On the other hand, antihypertensive agents can regulate the NLR [48]. For example, Fici et al., in their study, showed that a selective β1 blocker, nebivolol, can cause a reduction in blood pressure, vascular micro-inflammation prevention, and NLR reduction [49]. Likewise, in a study done by Karaman et al., it was found that valsartan, which is an Angiotensin II receptor blocker, reduces the NLR after 12 weeks of treatment more efficiently compared to amlodipine, a calcium channel blocker [50]. Moreover, the NLR reliably indicates the systemic inflammation status across the body [51].

The inflammation can possibly play a role in the pathophysiology of HTN through an increase in inflammatory markers like IL-1β, IL-6, and TNF-α [52]. Based on these findings, it is important to investigate the possible role of Neutrophils and lymphocytes in inflammation causing HTN.

Neutrophils, the predominant leukocyte in the blood, are polymorphonuclear granulocytes that play an important role in modulating innate and adaptive immune responses [48, 53]. In a study by Sela et al., the number of neutrophils was found to increase before the development of HTN in experimental models on mice [54]. Moreover, in another paper by Tatsukawa et al., it has been shown that the neutrophil count was remarkably high in hypotensive Japanese women compared to the control group [55]. Different studies indicate that isolated neutrophils surges are seen in arterial hypertension (AH) pre-clinical models, hypertensive individuals, and women with preeclampsia. These conditions increase levels of ROS as well as phagocytic activity. During host-defense reactions, myeloperoxidase (MPO) and NADPH oxidase activation increase. This results in the formation of neutrophil extracellular traps constituted by DNA fibers and granule proteins. The neutrophils adhere to endothelial cells, which can increase cellular permeability and cause vascular dysfunction [48]. Furthermore, Nicholls et al. illustrated that neutrophils incubated by norepinephrine had an increased release of IL-6 and MPO [56]. This suggests a possible regulatory function for neutrophils dependent on the sympathetic system [48].

A study by Morton et al. strongly suggests a direct involvement of neutrophils in the control of blood pressure. They indicate that decreased neutrophils in normotensive mice can lead to a reduction in endothelial-dependent vasoconstriction and systolic blood pressure [57].

Therefore, according to these aforementioned mechanisms, increased neutrophil counts can likely attribute to high blood pressure.

The leukocyte response seen in increased NLR ratios is lymphocyte dependent. Reduction in the number of lymphocytes results in physiologic stress and poor health status [58]. There are various subtypes of T lymphocytes that affect blood pressure by regulating cytokine release throughout the cardiovascular system [59]. Zhang et al. found that T-bet deficient mice were unable to initiate a T_h_1 response. These mice had sustained hypertensive responses but were protected from renal damage from chronic angiotensin II provocation [60]. The data sugges that T_h_1 cells can cause kidney injury that is independent of high blood pressure [59].

Secretion of IL-17, known as a proinflammatory cytokine, is primarily by T_h_17 cells. This release plays a role in the pathogenesis of many autoimmune diseases. The effect of T_h_17 lymphocytes on blood pressure is still controversial. However, the injurious effect of IL-17 or IL-23 deficiency in the DOCA/salt model of hypertension indicates a protective role for T_h_17 cells [59].

In opposition to the inflammatory role of T_h_1 and T_h_17 cells, regulatory T lymphocytes can modulate the anti-inflammatory cellular immune responses [58, 59]. An animal study by Barhoumi et al. showed that T_reg_ cells by mediating the angiotensin II response [61]. T_reg_ cells produce IL-10, which is an important cytokine. In addition to immunosuppression, endogenous IL-10 can reduce oxidative stress and vascular dysfunction by a blood pressure-independent mechanism. It has been shown that exogenous IL-10 can reduce blood pressure to the normal range and make the endothelial function normal in hypertensive pregnant mice [59, 62]. Thus, the protective effect of T_reg_ cells mediated through the IL-10 response warrants further investigation.

Despite the fact that additional studies should be done to elucidate the role of CD8 + T Lymphocytes in modulating hypertension, it has been shown that mice deficient in transcription factor inhibitor of differentiation (Id2) have altered CD8 + T cell memory and decreased natural killer cells. These mice do not exhibit hypertension induced by angiotensin II [59].

In summary, different subtypes of T lymphocytes can induce various levels of inflammation, which can either lead to hypertension or protect against it. The protective role of Treg cells was indicated, and it was stated that Th17 might have some protective effect against HTN if appropriately regulated.

Another type of lymphocyte is B cells which are necessary for adaptive immunity. The mechanisms by which B cells can play a role in hypertension has not been explored enough. However, it has been indicated by Chan and colleagues that angiotensin infusions induce further increase in B cells as well as plasma cell activation in lymphoid tissues. On the other hand, anti-CD20 antibody administration and genetic deficiency of B cells can cause the protection of mice against the hypertensive effects of angiotensin II [63]. Finally, additional studies focusing on the role of B cells in hypertension are needed to investigate novel mechanisms.

In our study, we indicate that in hypertensive patients, the number of neutrophils is increased, and the number of T lymphocytes that have a protective role is decreased. So, the NLR will be higher in hypertensive individuals and lower in normative controls.

As it has been stated before that non-dipper hypertensive individuals have a higher cardiovascular disease risk due to myocardial infarction and target organ damage compared with dipper hypertensive patients [51, 64]. These conditions are thought to be due to high platelet activity and increased inflammation [65]. The higher NLR levels in non-dipper hypertensive patients than dipper patients can indicate an increased pro-inflammatory state [64]. Moreover, it has been illustrated that the NLR can be used to independently predict long-term mortality and myocardial infarction [51]. Bayrakci et al. showed that the platelet-to-lymphocyte ratio (PLR), which is considered an inflammatory marker, is also remarkably higher in non-dipper hypertensive patients compared to dipper ones [66]. Inflamed tissues secrete some cytokines like IL-6, which contribute to vascular dysregulation. Through the influence of the cytokines, as mentioned earlier, the liver synthesizes C-reactive protein (CRP). High CRP levels can damage vessel walls. Also, there is an association with increased serum uric acid levels. This has been increased with higher cardiovascular disease [65]. Systemic inflammation can cause bone marrow dysfunction, leading to varied red blood cell size. Increased red blood cell distribution width (RDW) may be seen in inflammation [67]. Interestingly, CRP, uric acid, and RDW values are significantly higher in the non-dipper hypertensive patients compared with the dipper hypertensive patients and control group [65].

Higher blood pressure levels in non-dipper hypertensive individuals over the night can cause endothelial damage that triggers the proinflammatory process. Furthermore, inflammation can lead to blood pressure elevation. As a result, increased inflammation and high blood pressure both can feedback on each other contributing to cellular damage [65, 68]. Finally, because non-dipper hypertensive individuals have higher blood pressure during the night, they have increased inflammation which increases mortality and morbidity [65].

### Limitation

Some limitations of our study do exist. First, geographic variability is essential to consider in the context of these results. The majority of current studies on this topic were performed in Turkey. Disparities in both HTN rates as well as HTN outcomes have been shown within different geological locations. It is important to note that the results from the studies on this topic to date may not be as applicable to hypertensive patients located in different geographical regions. Thus, similar prospective and retrospective studies are warranted in wider geographic locations to characterize any potential differences between these populations. Second, heterogeneity in studies was greater than expected due to various treatment regimens, age ranges, and gender differences for included patients. Therefore, widespread validity is a concern, and future larger prospective studies are needed. Third, this review was not registered in PROSPERO. Finally, several studies are limited by bias, whether based on selection or publication, which should be considered.

## Conclusion

The current study is mainly providing knowledge of pathology of hypertension. Patients with HTN had higher level of NLR than normotensive individuals. In addition, patients with non-dipper HTN had higher NLR than those with dipper HTN. NLR represents a unique inflammatory marker whose elevation in HTN provides implications regarding immune system imbalance in the pathogenesis of the disease. In evaluation of included studies, it can be concluded that there may be association between HTN and NLR. Ultimately, with the development of new biomarkers and therapeutic modalities, we can better prevent and treat delirium to decrease long-term morbidity and mortality.

## Data Availability

The dataset supporting the conclusions of this article is included within the article.

## Abbreviations

NLR: Neutrophil to lymphocyte ratio
PLR: Platelet to lymphocyte ratio
WMD: Weighted mean difference
95% CI: 95% Confidence interval
CRP: C-reactive protein
HTN: Hypertension
VEGF: Vascular endothelial growth factor
MPO: Myeloperoxidase
CRP: C-reactive protein
RDW: Red blood cell distribution width
ND: Not Declared
CCB: Calcium channel blocker
ACE: Angiotensin-converting enzyme
ARB: Angiotensin receptor blockers
